# A single-nucleus transcriptomic characterization of *EGFR* G719X/S768I double-mutant lung adenocarcinoma identifies an immunosuppressive niche defined by *COL4A3*-*CD44* interaction

**DOI:** 10.1016/j.tranon.2026.102951

**Published:** 2026-07-31

**Authors:** Chao Zhang, Lin Liu, Ruoyu Deng, Jialing Lv, Chenghao Mei, Wen Zhang, Liuqing Yang, Fang Yang, Runxiang Yang, Yupeng Cun

**Affiliations:** a Department of the Second Medical Oncology, The Third Affiliated Hospital of Kunming Medical University, Kunming, Yunnan 650118, China; b Pediatric Research Institute, Chongqing Key Laboratory of Child Neurodevelopment and Cognitive Disorders, National Clinical Research Center for Children and Adolescents' Health and Diseases, Ministry of Education Key Laboratory of Child Development and Disorders, Children’s Hospital of Chongqing Medical University, Chongqing, 400014, China; c Department of Oncology, Qujing Central Hospital of Yunnan Province / Kunming Medical University Affiliated Qujing Hospital, Qujing, Yunnan, 655000, China

**Keywords:** *EGFR double mutations*, Lung adenocarcinoma, Single-nucleus RNA sequencing, EMT-like malignant, *COL4A3*-*CD44* interaction

## Abstract

*EGFR* double mutations (G719X/S768I) represent a clinically recalcitrant subtype of lung adenocarcinoma (LUAD) characterized by diminished tyrosine kinase inhibitor (TKI) sensitivity. However, the specific tumor microenvironment (TME) features and intercelluar crosstalk driving this therapeutic resistance poorly defined. We performed single-nucleus RNA sequencing (snRNA-seq) on formalin-fixed paraffin-embedded (FFPE) tissues from six *EGFR* G719X/S768I double-mutant (EGFR_double) and five no *EGFR* mutation (Non_EGFR) LUAD patients. To provide a comprehensive reference, we integrated our FFPE data with publicly fresh-tissue single cell RNA sequencing (scRNA-seq) data with seven *EGFR* single-mutant LUAD (EGFR_single) and five adjacent normal (Adjacent), generated a comprehensive atlas of 246,086 cells. Findings were further validated using multiplex immunohistochemistry (mIHC) and pharmacogenomic profiling.

We identified a specialized EMT-like malignant subpopulation (MP4) uniquely enriched in EGFR_double, which exhibits a distinct lipid-glycosylation metabolic profile that stabilizes *CD44* expression. Cell-cell communication analysis revealed enhanced *COL4A3*-*CD44* interactions between EMT-like malignant MP4 cells and metabolically reprogrammed, immunosuppressive alveolar macrophages in EGFR_double compared with Non_EGFR. MIHC confirmed in situ proximity of this significantly increased *COL4A3*-*CD44* co-localization in EGFR_double. Furthermore, drug sensitivity analysis identified specific vulnerabilities of this subtype.

Our study identifies a previously unrecognized malignant-myeloid niche and TME in EGFR_double. These findings provide a translational framework for identifying a potential novel therapeutic targets and suggest that combinatorial strategies targeting the *COL4A3*-*CD44* interaction may overcome therapeutic recalcitrance in this challenging clinical population.

## Highlights

• A single-nucleus transcriptomic of FFPE tissues reveals distinct TME features in *EGFR* G719X/S768I LUAD.
• EMT-like malignant subtype with lipid metabolic programs is enriched.
• Alveolar macrophages show metabolically stressed, immunosuppressive programs.
• COL4A3-CD44 crosstalk defines malignant-macrophage interaction niche.

## Introduction

Lung adenocarcinoma (LUAD) is the most common histological subtype of lung cancer, accounting for approximately 40% of all lung cancer cases and representing a leading cause of cancer-related mortality worldwide [1]. *EGFR* mutations are the most prevalent driver alterations in LUAD, particularly among East Asian populations, with mutation rates reaching 40–55% [[2], [3], [4]]. *EGFR* tyrosine kinase inhibitors (TKIs) have revolutionized the treatment of *EGFR*-mutant LUAD, with classical mutations (exon 19 deletions and L858R) demonstrating response rates of 60–70% [5]. However, rare *EGFR* mutations, including compound mutations involving G719X, L861Q, and S768I, exhibit substantially different clinical outcomes that warrant further investigation.

Among rare *EGFR* variants, G719X and S768I co-mutation constitutes a clinically distinct and significant subtype, comprising ∼5% of all *EGFR*-mutant LUAD cases [[6], [7], [8]]. Clinical evidence showed such patients harboring G719X/L861Q/S768I compound mutations exhibit markedly inferior objective response rates (41.6%vs. 66.5%) and abbreviated progression-free survival (7.7 vs. 11.4 months) compared to those with classical mutations [9], while another study explored treatment strategies for *EGFR* double-mutant LUAD [10]. ScRNA-seq studies underscores that how TME shape therapeutic outcomes in *EGFR*-mutant lung cancer, which reveal different *EGFR* mutation types are associated with distinct TME and functional states [[11], [12], [13]]. Notably, a recent study demonstrated that *TP5*3 co-mutations in LUAD reshape TME through *SPP1*^+^ macrophage-fibroblast niches that facilitate tumor progression [14]. Similarly, integrative spatial and scRNA-seq analyses identified *BCL2L1* upregulation as a key mediator of TKI resistance [12]. Recent advances in FFPE-based snRNA-seq enable profiling from FFPE tissues, expanding the accessibility of single-cell analyses to archival clinical specimens [15]. While cell-recovery patterns differ between FFPE and fresh tissues [16], well processed FFPE snRNA-seq enables robust, biologically meaningful comparisons. However, how *EGFR* G719X/S768I double mutations orchestrate immunosuppression niches with TME remain elusive, representing a critical gap in precision oncology.

To characterize the TME and delineate core functional modules of *EGFR* double-mutant LUAD, we performed snRNA-seq profiling of eleven FFPE tissues of *EGFR* G719X/S768I double-mutant and *EGFR* wild-type LUAD patients, which we subsequently integrated with publicly scRNA-seq data from single-mutant *EGFR* and adjacent normal as comparison. Our analysis identified a specialized EMT-like malignant subpopulation that orchestrates an immunosuppressive niche via a *COL4A3*-*CD44* signaling interaction with alveolar macrophages, which further validated via multiplex immunohistochemistry (mIHC). Collectively, our study identified a previously unrecognized *COL4A3*-*CD44* linked metabolic-myeloid niches as a potential driver of TME-mediated immunosuppression, suggesting a potential novel therapeutic resistance in the *EGFR* G719X/S768I double-mutant LUAD.

## Materials and methods

### Patients

Six EGFR_double and five Non_EGFR FFPE of LUAD patients (Table S1) were collected from Qujing First People’s Hospital with informed patients and approved by the Ethics Committee of Qujing First People’s Hospital (No. 2024– 076– 02). *EGFR* single mutation and adjacent normal samples were sourced from the National Genomics Data Center [11] (Table S2). Bulk RNA-sequencing data for TCGA-LUAD [17] and East Asian (EAS) samples [18] were downloaded from the data availability sections of the respective publications, while whole-exome sequencing (WES) and RNA-sequencing data for samples from the Xuanwei (XW) region were obtained from the National Genomics Data Center (HRA000124) [19].

### Single-cell and -nucleus RNA sequencing for FFPE tissue and data processing

FFPE tissues were prepared in supplementary methods, and then sequenced by 10X barcode gel beads, and oil were loaded separately into designated wells of the Chromium Next GEM Chip Q (1000,418, 10X Genomics), and Gel Beads-in-Emulsion (GEMs) were generated using the Chromium X system (10X Genomics). Per-sample quality metrics including number of cells, number of reads, median UMI counts per cell, mean reads per nucleus, and median genes per nucleus are provided in Table S3. Detailed sequencing steps were described in the Supplementary Methods.

Raw data of FFPE snRNA-seq were processed with Cell Ranger multi (v7.0.0), quality control was processed by Seurat (v4.4.0) [20], which evaluated total UMIs, detected genes, and mitochondrial content, and DoubletFinder (v2.0.3) was used to remove doublets. A public dataset was processed in similar way, and dataset integration, dimensionality reduction and cell types annotation were described in the supplementary methods. The final integrated dataset comprised 246,086 cells from both FFPE snRNA-seq and public scRNA-seq dataset, and were visualized via Uniform Manifold Approximation and Projection (UMAP).

### Copy number variation based malignant cell inference and clustering analysis

InferCNV (v1.18.1) [21] was used to infer copy number variation (CNV) profiles of single nucleus/cell transcriptomes by detecting large-scale chromosomal gains and losses. Fresh and FFPE datasets were analyzed separately, where B cells were used as normal reference. CNVs computation process was described in supplementary method, malignant cells identified by CNV profiles.

### Transcriptional module identification via genenmf

Cells identified as malignant and originating from tumor tissue were grouped by sample for non-negative matrix factorization (NMF) using GeneNMF (v0.6.0) [22]. For each sample, NMF was performed with a specified rank range of k = 4 to 9, using the top 2000 highly variable genes as input features, *multiNMF* function was applied to perform decomposition across samples, followed by clustering of extracted programs using *getMetaPrograms*, with nMP set to 9 to define unified transcriptional modules. Stability of each module was assessed through Jaccard similarity analysis with a cutoff of 0.8. Finally, *plotMetaPrograms* was used to visualize module composition and conservation.

### Single-cell and -nucleus metabolic activity analysis

We evaluate metabolic activities of snRNA-seq data using scMetabolism [23]. Metabolic gene sets were obtained from the Kyoto Encyclopedia of Genes and Genomes (KEGG) database, and pathway activity was scored using *AUCell* method. Results were visualized using pheatmap (v1.0.12), with row-wise normalization applied to highlight differences in metabolic states across cell types.

### Single-cell and -nucleus transcription factor inference

PySCENIC (v0.11.2) [24] was applied to infer and cluster single-cell regulatory networks in malignant functional modules and myeloid cell subpopulations. Raw count matrices were used as input. First, *pyscenic grn* module was applied to infer co-expression networks between transcription factors (TFs) and their potential target genes. Next, *pyscenic ctx* module utilized a motif database to identify enriched motifs and define regulons. Finally, *pyscenic aucell* module computed regulon activity scores at single-cell resolution.

### RNA velocity and state transition analysis

ScVelo (v0.2.4) [25] was applied to infer trajectory in snRNA-seq. Data normalization and neighborhood graph computation were performed, and followed by estimation of transcriptional dynamics using *dynamical* model to infer RNA velocity vectors for each cell. Dynamic cell state trajectories across different mutation types were visualized on the UMAP embedding using *velocity_embedding_stream*.

### Pathway activity and enrichment analysis

Gene set variation analysis (GSVA, v1.46.0) [26] was applied to assess pathway activity in alveolar macrophage subtypes from EGFR_double and Non_EGFR. Gene expression averages were calculated using *AverageExpression* after filtering low-expressed genes and standardizing expression values. Gene sets for gene ontology biological processes (GO-BP) and KEGG pathways were computed with *gsva* function. Differential pathway analysis between the two groups was conducted using limma (v3.54.2) [27], as described in the supplementary methods. To quantify infiltration of malignant subtypes and alveolar macrophages in TCGA-LUAD and EAS bulk RNA-seq datasets, we performed ssGSEA with subtype-specific marker genes, generating enrichment scores for each cellular subset.

MSigDB database (described in supplementary method) was employed to evaluate pathway activity in macrophage subtypes. Pathway module scores were calculated using *AddModuleScore* function, which computes difference between average expression of target genes and that of 100 control genes for each cell.

PROGENy (v1.24.0) [28] was used to infer and standardize pathway activity scores across various cell types. High-activity pathways were defined as those exceeding 85% of mean activity values across all pathways.

### Integrated cell-cell communication in sn/scRNA-seq data

CellChat (v1.6.1) [29] was employed to characterize intercellular communication networks between EGFR_double and Non_EGFR, with emphasis on interactions involving malignant cells and myeloid subtypes. Differential ligand-receptor and co-factor interactions were identified using significance threshold of p = 0.05, ligand.logFC = ±0.25, and receptor.logFC = ±0.25.

Subsequently, NicheNet [30] was applied to investigate the downstream target genes potentially regulated by the *COL4A3-CD44* interaction. Alveolar macrophages cell population was defined as the signal-receiving cell type, with EGFR_double designated as the disease group and Non_EGFR as the control group. Differential expression analysis was conducted using the FindMarkers function, with a logFC threshold = 0.25 and a minimum expression proportion of 10%.

Genes significantly upregulated in the disease group were further selected based on an adjusted *p* value < 0.05 and an average log2 fold change (avg_log2FC) > 0. To ensure compatibility with downstream NicheNet analysis, only genes present in the ligand-target regulatory matrix were retained, thereby defining the receiver-specific gene set of interest (geneset_oi).

*COL4A3* was then selected as the ligand of interest, and its potential target genes within the predefined geneset_oi were identified. For each candidate target gene, the corresponding NicheNet regulatory potential score was extracted, and the Pearson correlation between target gene expression and *CD44* expression levels was calculated. Finally, the NicheNet regulatory potential and *CD44* correlation were integrated by multiplying the two metrics to generate an integrated score, which was used to rank downstream target genes associated with the *COL4A3-CD44* interaction signaling.

### Drug sensitivity prediction using OncoPredict

OncoPredict [31] was employed to predict drug sensitivity using the Genomics of Drug Sensitivity in Cancer version 2 (GDSC2) dataset. EAS count data and XW count data were normalized to transcripts per million and log₂-transformed (log₂[x + 1]), with batch correction (batchCorrect = "eb") applied to enhance compatibility between training and test datasets. Predicted drug-sensitivity scores for EGFR_double (G719X/S768I) (Table S4) were analyzed for associations with *CD44* and *COL4A3* expression using Spearman correlation.

### Multiplex immunohistochemistry

FFPE tissue sections (5 μm) from seven LUAD patients (4 EGFR_double and 3 Non_EGFR) were subjected to multiplex immunohistochemistry. Sections were incubated overnight at 4 °C with primary antibodies against CD68 (Huabio, ER1901– 32, 1:1000 dilution), COL4A3 (Proteintech, AP61199, 1:500 dilution), CD44 (Abways, CY5138, 1:500 dilution), and pan-cytokeratin (Abcam, ab7753, 1:1000 dilution), followed by tyramide signal amplification (Reakai Biotech, RC0086Plus-67RM). All antibodies were tissue-validated prior to use. Three high-quality regions per section (17 regions total) were semi-quantitatively assessed for co-localized signals and the proportion of CD68⁺ cells within co-localized regions. Statistical comparisons between EGFR_double and Non_EGFR were performed using the Wilcoxon test.

## Results

### Integrated single nucleus and cell transcriptomic landscape reveals distinct TME in *EGFR* G719X/S768I double-mutant LUAD

**Fig. 1:**
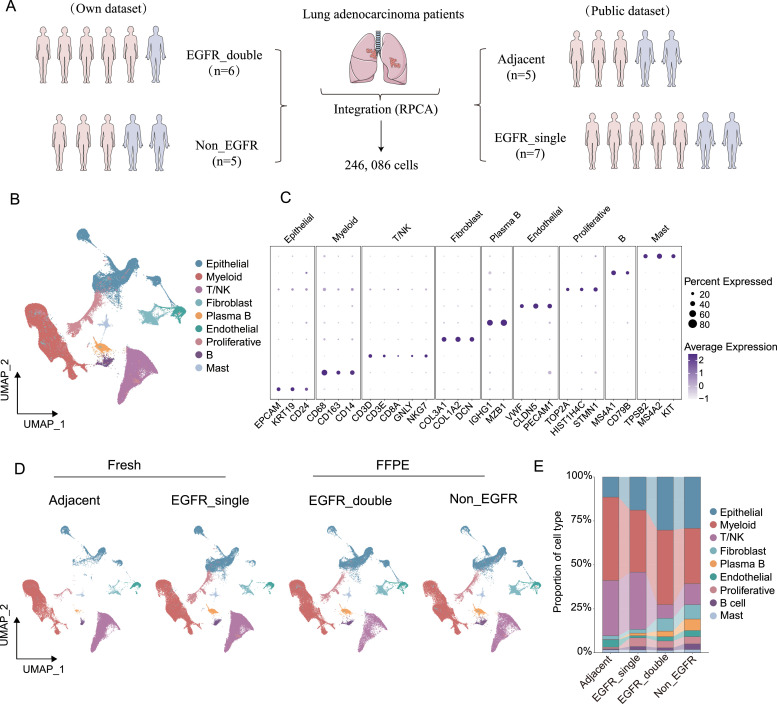
Single nucleus and cell transcriptome landscape of distinct *EGFR* mutation subtypes in LUAD. Schematic overview of the experimental design, illustrating the LUAD cohorts analyzed, including in-house EGFR_double and Non_EGFR, as well as public datasets of adjacent normal tissues and EGFR_single. B. UMAP of nine cell types. C. Marker genes for each cell type annotation, with dot size indicating the proportion of expressing cells and color intensity representing average expression levels. D. UMAP plots of different mutation groups. E. Relative proportions of cell types in each mutation type.

To delineate the cellular architecture of *EGFR* G719X/S768I double-mutant LUAD, we established a comprehensive single-nucleus/cell transcriptomic atlas by integrating in-house snRNA-seq data from 11 FFPE specimens (6 EGFR_double and 5 Non_EGFR) with publicly available fresh tissue scRNA-seq datasets (5 adjacent normal and 7 EGFR_single samples). After quality control and batch effect mitigation via reciprocal principal component analysis (RPCA), we generated 246,086 high-quality cells for downstream analysis (Fig. 1A, S1A, B). Unsupervised clustering identified nine major cell types: epithelial, myeloid, T/NK, fibroblast, plasma B, endothelial, proliferative, B, and mast cells (Fig. 1B, C). Analysis of cell type proportions revealed significant heterogeneity across mutation types and tissue sources (Fig. 1D). Consistent with established benchmarks for archival specimen sequencing [16], we observed that FFPE samples exhibited distinct recovery signatures, characterized by reduced T/NK cell detection but enriched representation of epithelial and endothelial compartments compared to fresh tissues (Fig. 1E).

To account for these inherent technical biases, we implemented a stratified analytical framework: primary comparative conclusions were derived from within-platform comparisons (EGFR_double vs. Non_EGFR within the FFPE), while fresh tissue data served as an external reference for lineage annotation and validation of mutation-conserved features. Notably, within our FFPE data, EGFR_double exhibited a significantly elevated abundance of myeloid cells compared to Non_EGFR. This preferential immune infiltration suggested a mutation-specific remodeling of the myeloid niche, which warranted further investigation into its functional implications.

### Lipid-glycosylation metabolic coupling influence EMT-like programs and aggressive phenotypes in *EGFR* double-mutant LUAD malignant cells

**Fig. 2:**
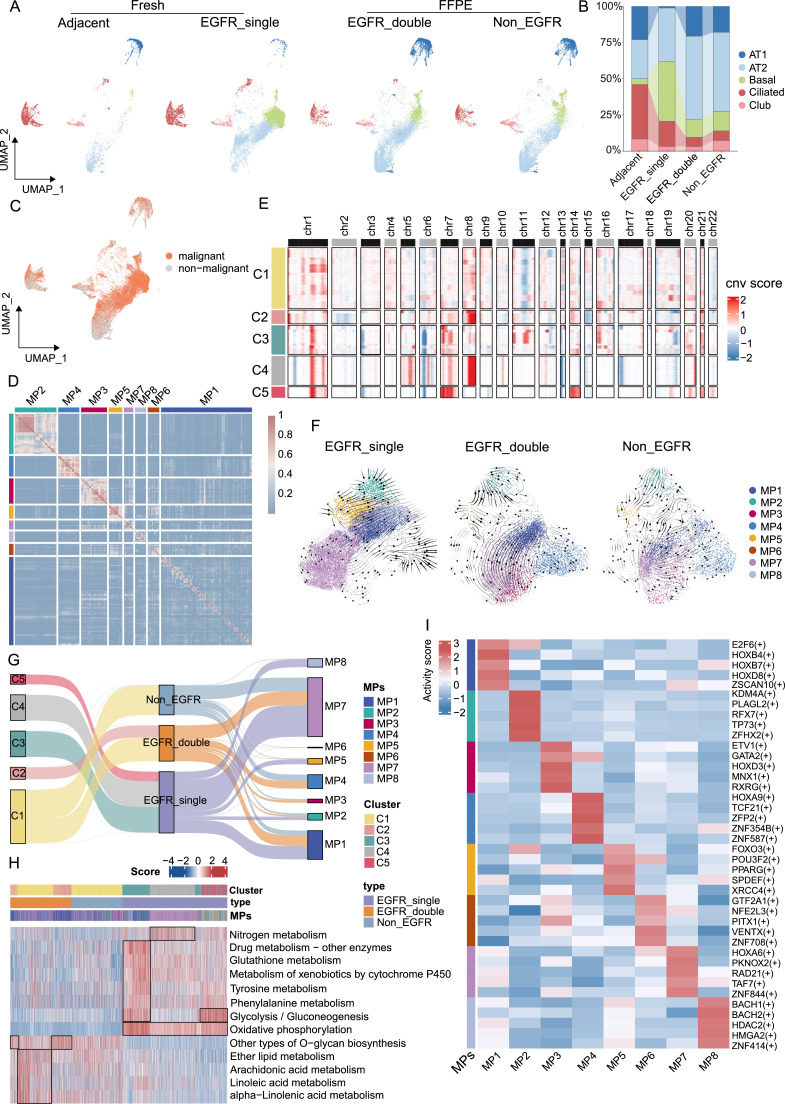
Functional modules, CNV subclones, and metabolic profile of epithelial subtypes for different *EGFR* mutation type in LUAD. UMAP of epithelial subtypes with different EGFR mutation type. B. Relative proportions of epithelial subtypes in different mutation type. C. UMAP of malignant (orange) and non-malignant (gray) epithelial cells. D. GeneNMF identified seven functional modules, where each column representing a cell and each row a gene, and color intensity indicates scaled expression levels. E. UMAP of RNA velocity analysis for EGFR_single, EGFR_double, and Non_EGFR, with arrows indicating the inferred direction of cell state transitions and colored by GeneNMF modules. F. Copy number variation profile derived from hierarchical clustering identified five subclones, where orange colored indicates amplification and blue indicates deletion across chromosomes. G. Sankey diagram illustrating the distribution of cells across five CNV subclones, mutation types, and eight functional modules. Flow width represents cell number, and node color denotes category. H. Heatmap of metabolic pathway scores across 5 subclones, mutation types, and 9 functional modules, where higher score indicated by deeper red. I. Heatmap the top five most active transcription factors inferred by PySCENIC in each malignant module, color intensity reflects regulon activity.

Re-clustering analysis of epithelial cells identified five subtypes: type I alveolar (AT1), type II alveolar (AT2), basal, ciliated, and club cells (Fig. S1D, E). AT2 cells predominated in both EGFR_double and Non_EGFR, consistent with the known cellular origin of LUAD (Fig. 2A, B). Malignant cells were identified based on copy number variation (CNV) profiles inferred from snRNA-seq data (Fig. 2C, S2A). We identified eight transcriptional modules in malignant cells via GeneNMF analysis, where MP1 (extra cellular matrix, ECM remodeling), MP2 (Cilium motility), MP3 (Inflammatory), MP4 (epithelial-mesenchymal transition-like, EMT-like), MP5 (Secretory), MP6 (Stress), MP7 (AT2-like), and MP8 (Hypoxia) (Fig. 2D, S2B, C, Table S5– 7). RNA velocity analysis delineated distinct mutation-specific lineages, where EMT-mediated transition from MP4 toward ECM-remodeling MP1 in EGFR_double, while Non_EGFR exhibited more diverse trajectories originating from AT2-like (MP7) states (Fig. 2E). CNV-based clustering delineated five distinct subclones (C1-C5), with C2 and C4 exhibiting chromosome 8 amplifications and C5 showing chromosomes 7 and 14 amplifications, where C1 was distributed across subtypes, C3-C5 predominated in EGFR_single, and C2 was uniquely to the EGFR_double (Fig. 2G, F, S2A).

Metabolic characterization revealed a distinct divergence: EGFR_single prioritize energy and antioxidant metabolism, whereas EGFR_double and Non_EGFR are enriched in lipid metabolism and glycosylation pathways. EMT-like (MP4 and C1) subpopulation exhibits a profound lipid-EMT metabolic coupling, marked by enhanced ether lipid and arachidonic acid metabolism in the EGFR_double (Fig. 2H). This metabolic signature likely drives increased invasiveness via membrane remodeling and pro-inflammatory signaling. Notably, the associated O-glycan biosynthesis serves as a functional switch for *CD44* regulation, priming these malignant cells for subsequent crosstalk within the immunosuppressive niche. Recent studies showed lipid-EMT metabolic coupling enhanced invasiveness and therapeutic resistance [32,33], and enrichment of O-glycan biosynthesis, a pathway critical for modulating the glycosylation status and functional stability of *CD44* [34,35]. MP4 module exhibited robust activation of Hedgehog and TGF-β signaling, alongside the upregulation of EMT-associated transcriptional regulators HOXA9 and TCF21 (Fig. 2I, S2B-D) [36,37]. MP4 was enriched in EGFR_double and Non_EGFR (Fig. 2D, S2E) identifies it as a conserved malignant driver, warranting a detailed comparative analysis of its mutation-specific functional states.

Altogether, these findings demonstrate that EGFR_double harbors a specialized EMT-like malignant subtype, which defined by a unique lipid-glycosylation metabolic feature that suggest its aggressive phenotype and primes tumor ecosystem for subsequent immunosuppressive remodeling.

### Divergent transcriptional programs and the *CD44*-mediated interface orchestrate myeloid remodeling in *EGFR* double-mutant LUAD

**Fig. 3:**
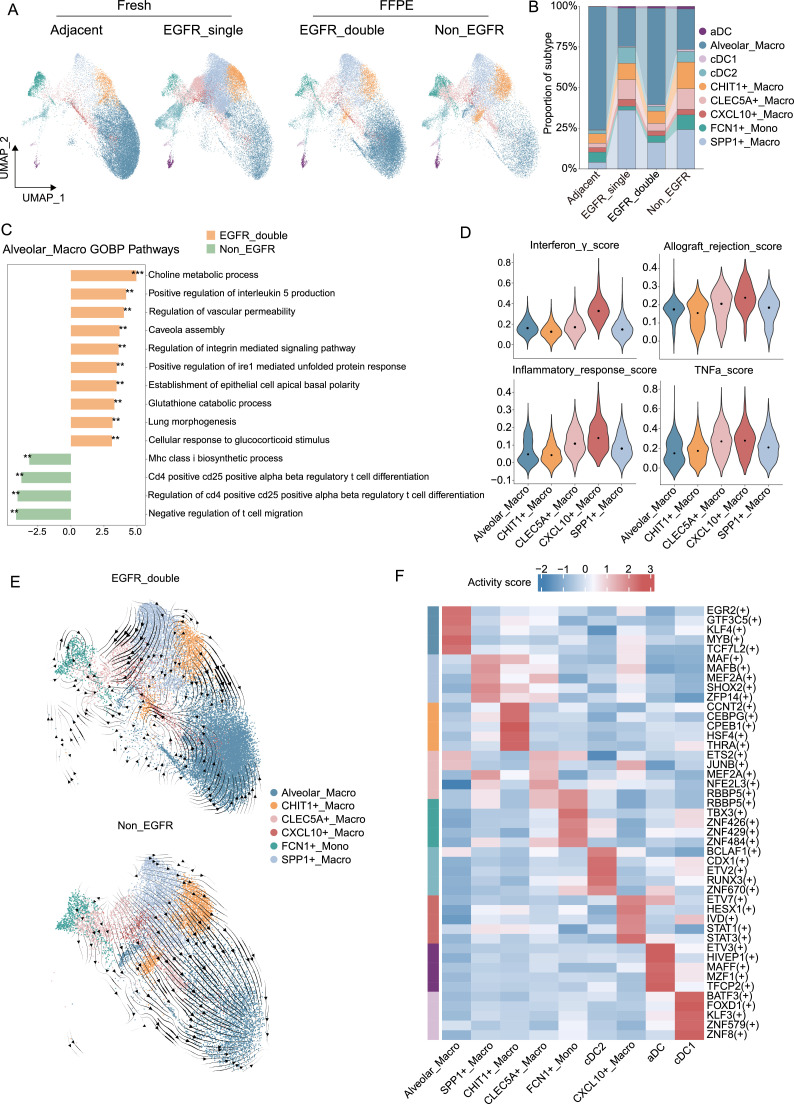
Identification and characterization of myeloid cell subtypes for different *EGFR* mutation type in LUAD. UMAP of myeloid subtypes across different mutation types. B. Bar plot depicting the relative proportions of myeloid subtypes across mutation groups. C. GSVA analysis of GO-BP in AMs comparing EGFR_double and Non_EGFR; orange indicates EGFR_double and green indicates Non_EGFR. **p < 0.01, ***p < 0.001. D. Pro-inflammatory gene signature scores across different macrophage subtypes, each dot represents the median value. E RNA velocity analysis for EGFR_double and Non_EGFR, visualized by UMAP and colored by macrophage subtypes, arrows indicate the inferred direction of cell state transitions. F. Heatmap showing the top five most active transcription factors in each myeloid subtype, redder colors indicate higher regulon activity.

Re-clustering analysis of myeloid cells identified nine subtypes: Alveolar macrophages (Alveolar_Macro, AM), *CHIT1*⁺ macrophages (CHIT1⁺_Macro), *CLEC5A*⁺ macrophages (CLEC5A⁺_Macro), *CXCL10*⁺ macrophages (CXCL10⁺_Macro), *SPP1*⁺ macrophages (SPP1⁺_Macro), activated dendritic cell (aDC), type 1 conventional dendritic cells (cDC1), type 2 conventional dendritic cells (cDC2), and *FCN1*⁺ monocytes (FCN1⁺_Mono) (Fig. S3A, B). The myeloid landscape exhibited distinct mutation-dependent remodeling, characterized by a significant enrichment of alveolar macrophages EGFR_double relative to Non_EGFR (Fig. 3A, B). While Alveolar_Macro maintain pulmonary equilibrium, our functional profiling reveals distinct mutation-specific reprogramming [38,39]. In EGFR_double, Alveolar_Macro are predominantly enriched to stress adaptation, metabolic regulation, and immune modulation pathways, contrasting with the inflammatory and T-cell regulatory programs observed in the Non_EGFR (Fig. 3C, S3C, Table S8– 9). This functional divergence leads to different prognostic results in two TCGA and EAS datasets, whereas high Non_EGFR AMs have significantly survival in the TCGA, EGFR_double AMs exhibit a trend toward poorer outcomes in the EAS (Fig. S3D, E). Collectively, these results indicate that EGFR_double functionally reshape the AM compartment to sustain a metabolically stressed and pro-tumorigenic niche.

Transcription factor activity inference delineated distinct regulatory pattern across mutation groups. EGR2 and KLF4 activities of AMs were higher in EGFR_double, which suggest to drive stress-responsive and pro-tumorigenic programs in former studies [[40], [41], [42]]. While homeostatic regulator TCF7L2 was notably reduced in EGFR_double. Furthermore, CXCL10⁺_Macro displayed prominent STAT3 signaling in EGFR_double, whereas ETV7 and STAT1 dominant signatures observed in EGFR_single (Fig. 3F, S3F).

Notably, *CD44* was highly expressed in both AMs and CXCL10⁺_Macro, with significantly elevated levels in EGFR_double compared to Non_EGFR (Fig. S3G). This mutation-specific upregulation, coupled with the previously identified O-glycan metabolic reprogramming in MP4 cells, suggested a potential *CD44*-dependent signaling interaction orchestrating the crosstalk between malignant cells and the myeloid compartment.

### EMT-associated COL4A3-CD44 interaction serves as a primary signaling hub for malignant-myeloid interaction in EGFR_double LUAD

**Fig. 4:**
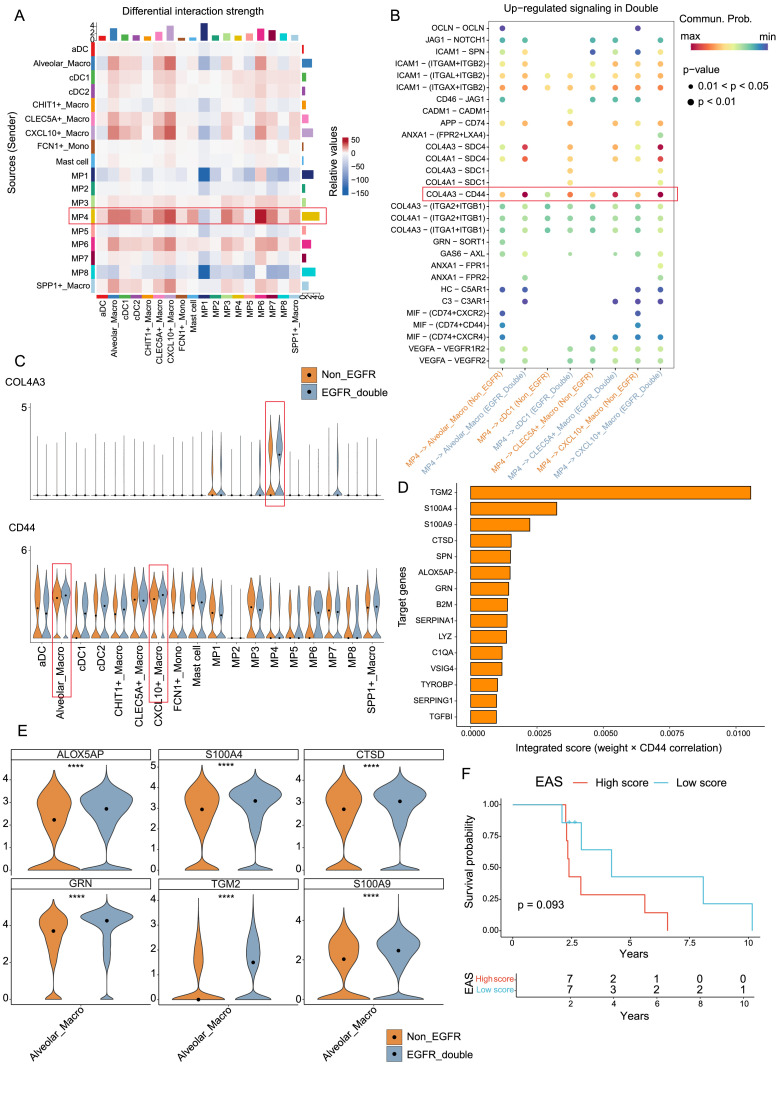
Cell-cell communication between malignant functional modules and macrophages in EGFR_double and Non_EGFR LUADs. Heatmap of differential communication strength between EGFR_double and Non_EGFR. Rows and columns represent sender and receiver cell types; color intensity reflects the magnitude of difference (red indicates enhanced communication in EGFR_double, blue indicates reduced). B. Upregulated ligand-receptor pairs in EGFR_double. Circle color reflects the communication probability between interacting cell populations (redder indicates higher), while size represents statistical significance (p-value). C. Expression of *COL4A3* and *CD44* in myeloid subclusters and malignant functional modules, compared between EGFR_double and Non_EGFR, each dot represents the median value. D. The x-axis represents the integrated score calculated by multiplying the NicheNet regulatory potential with the correlation coefficient of CD44 expression, and the y-axis indicates the predicted downstream target genes. E. Violin plots showing the expression differences of the six selected target genes in Alveolar_Macro cells between EGFR_double and Non_EGFR (*** *p* < 0.001). F. Survival analysis based on COL4A3-CD44-mediated gene signature scores in EGFR_double from the EAS dataset. High and Low denote groups with high and low gene signature scores, respectively.

To elucidate the molecular conduits driving myeloid reprogramming, we performed a cell-cell communication analysis focusing on the interaction between the MP4 (EMT-like) malignant modular and EGFR_double enriched macrophages. We found COL4A3-CD44 interaction emerged as the most prominent and specific interaction within the double-mutant microenvironment (Fig. 4A, B, S4A, B). Notable, *COL4A3* was highly expressed in MP4 (EMT-like) and significantly elevated in EGFR_double compared to Non_EGFR, while its cognate receptor, *CD44*, was predominantly expressed in Alveolar_Macro and CXCL10⁺_Macro (Fig. 4C, S4C, D).

To explore the downstream transcriptional output of this interaction, we integrated NicheNet regulatory potential with *CD44*-correlated expression scores (Fig. 4D, S4E). We identified a six-gene signature representing the transcriptional activation of *COL4A3-CD44* signaling, which was significantly upregulated in EGFR_double (Fig. 4E). Stratification based on this signature revealed a consistent survival trend: patients with high signature scores exhibited poorer prognosis in both the FFPE and the two bulk RNA-seq cohorts (Fig. 4F, S4F).

Altogether, these findings suggest that the *COL4A3-CD44* interaction serves as a functional bridge, translating tumor-intrinsic EMT programs into a systemic pro-tumorigenic myeloid response in EGFR_double.

### Multiplex immunohistochemistry validates of *COL4A3*-*CD44* co-localization in *EGFR* double-mutant LUAD

**Fig. 5:**
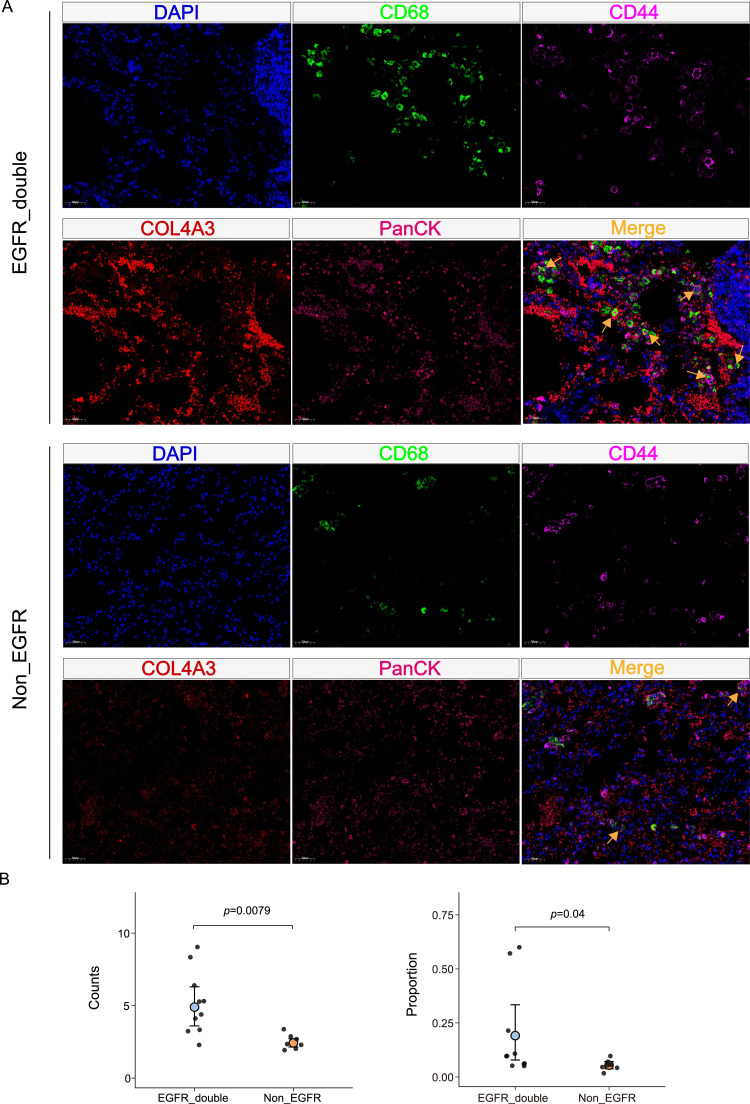
Functional validation of COL4A3 and CD44 in EGFR_double and Non_EGFR LUADs. Representative mIHC images showing expression and colocalization of CD68, CD44, COL4A3, and PanCK in EGFR_double and Non_EGFR. B. Semi-quantitative analysis of merged positive signals (left) and proportion of merged signals relative to CD68⁺ cells (right). EGFR_double: n = 10; Non_EGFR: n = 7. Statistical differences were evaluated using the Wilcoxon test.

To validate spatial evidence the predicted *COL4A3*-*CD44* interaction, we performed mIHC on our 11 FFPE LUADs. We specifically stained for markers identifying MP4 (PanCK and COL4A3), CD44⁺ macrophages (CD68 and CD44) to delineate their topographical distribution, which demonstrated distinct staining patterns between EGFR_double and Non_EGFR (Fig. 5A, S5– 6). Semi-quantitative analysis revealed that both the abundance of co-localized PanCK⁺/COL4A3⁺/CD68⁺/CD44⁺ signals and the proportion of CD68⁺ cells within co-localized regions were significantly increased in EGFR_double compared to Non_EGFR (Fig. 5B, Table S10– 11). Statistical analysis using the Wilcoxon test confirmed significant differences between groups (Counts: *p* = 0.0079, Proportion: *p* = 0.04). These results provide *is situ* experimental validation supporting enhanced *COL4A3*-*CD44* interactions between malignant cells and macrophages in EGFR*_*double.

To further assess the robustness of the *COL4A3*-*CD44* association, we analyzed three independent bulk transcriptomic cohorts, including TCGA-LUAD, an East Asian LUAD cohort (EAS), and the Xuanwei LUAD cohort. Across three datasets, *COL4A3* expression was consistently positively correlated with *CD44* expression (Fig. S7A, B). Our single-cell analysis focused specifically on *EGFR* G719X/S768I-mutant LUAD, we further evaluated all available Asian patients carrying this co-mutation in the Xuanwei and EAS cohorts. Despite limited cases owing to the rarity of this *EGFR* subtype, the strong positive correlation persisted (rho = 0.80, *p* = 0.0003, Fig. S7C), which further supporting the relevance of the *COL4A3*-*CD44* interaction in *EGFR* G719X/S768I-mutant LUADs.

### Therapeutic vulnerabilities linked to *COL4A3*-*CD44* interaction

To identify potential therapeutic targets associated with *COL4A3-CD44* signaling, we performed drug sensitivity profiling in EGFR_double. Elevated expression of both *CD44* and *COL4A3* significantly correlated with increased sensitivity (lower IC_50_) to a spectrum of antineoplastic compounds (Fig. S8A, B). Notably, we identified a core set of shared therapeutic vulnerabilities, including inhibitors targeting p38 MAPK (Doramapimod), PARP (Niraparib), IGF-1R (Linsitinib, BMS-754,807), BET (AZD5153), and JAK1 pathways (Fig. S8C, Table S12– 13). The consistent negative correlation between the expression of this signaling interaction and the IC_50_ values of these agents suggests that *COL4A3* and *CD44* may serve as predictive biomarkers for therapeutic response, offering novel pharmacological strategies to bypass resistance in EGFR_double.

### *EGFR* mutation specific functional biases in endothelial and fibroblast

**Fig. 6:**
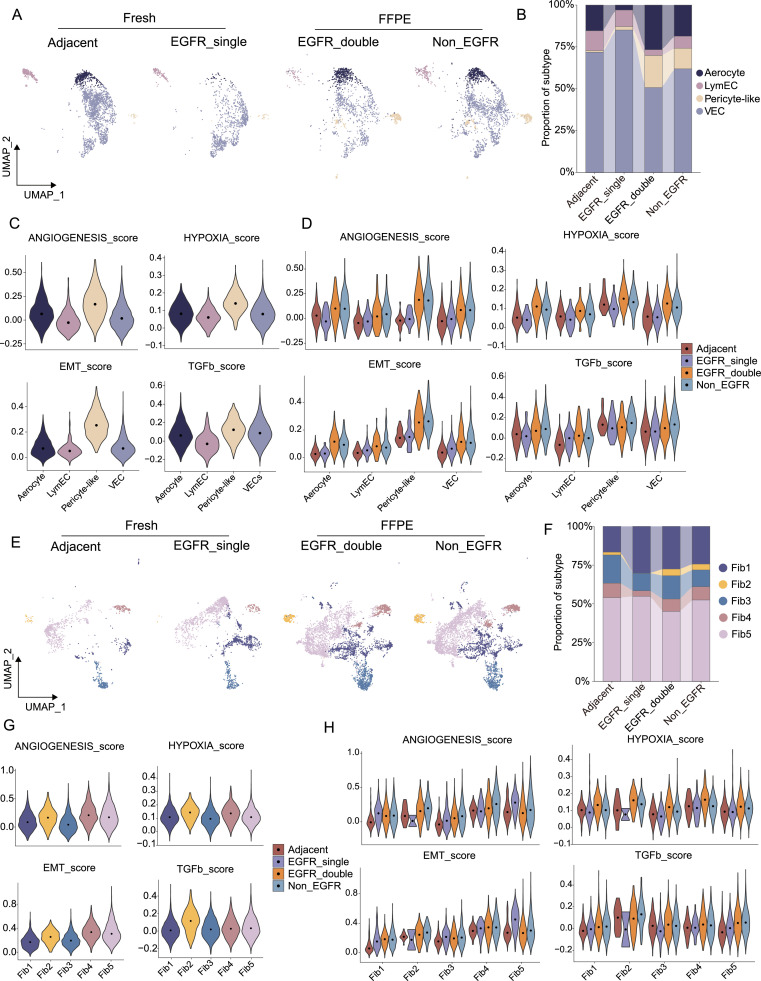
Identification and characterization of fibroblast and endothelial cell subtypes for different *EGFR* mutation type in LUAD. A. UMAP visualization of fibroblast subtypes across mutation subtypes. B. Bar plots displaying the relative proportions of fibroblast subtypes across mutation types. C. Signature scores for hypoxia, angiogenesis, EMT, and TGF-β pathways across fibroblast subtypes, each dot represents the median value. D. Comparison of hypoxia, angiogenesis, EMT, and TGF-β signature scores across fibroblast subtypes grouped by Adjacent, EGFR_single, EGFR_double, and Non_EGFR, each dot represents the median value. E. UMAP of endothelial subtypes across mutation subtypes. F. Bar plots displaying the relative proportions of endothelial subtypes across mutation types. G. Signature scores for hypoxia, angiogenesis, EMT, and TGF-β pathways across endothelial subtypes, each dot represents the median value. H. Comparison of hypoxia, angiogenesis, EMT, and TGF-β signature scores across endothelial subtypes grouped by Adjacent, EGFR_single, EGFR_double, and Non_EGFR, each dot represents the median value.

Re-clustering of endothelial cells (EC) identified four subtypes: Aerocytes, Lymphatic EC (LymEC), Pericyte-like, and Vascular EC (VEC) (Fig. S9A, B). EGFR_double tumors were characterized by an enrichment of Pericyte-like cells and Aerocytes, contrasting with the VEC-dominant profile of the EGFR_single. Functionally, Pericyte-like cells exhibited peak activity in angiogenesis and hypoxia in EGFR_double, whereas EMT and TGFβ signaling predominated in Non_EGFR (Fig. 6C, D).

Re-clustering of fibroblast identified five subtypes (FB1-FB5), including antigen presenting (FB1), endothelial-like (FB2), pericyte-like (FB3), progenitor-like (FB4), and myofibroblast (FB5) (Fig. S9C, D). Notably, FB2 was enriched in both EGFR_double and Non_EGFR subtypes. Functionally, FB4 demonstrated the strongest angiogenic and EMT potential, particularly in Non_EGFR. In contrast, FB2 showed a mutation-specific functional split: responding primarily to hypoxia in EGFR_double and to TGFβ signaling in Non_EGFR (Fig. 6G, H).

Collectively, these findings demonstrate that endothelial and fibroblast subtypes undergo mutation-specific functional reprogramming. The coordinated activation of hypoxia, angiogenesis, and TGFβ signaling across these stromal compartments suggests a divergent but synergistic contribution to tissue remodeling and metastatic facilitation across *EGFR* mutation types [43].

## Discussion

*EGFR* G719X/S768I double mutations represent a clinically challenging subtype of LUAD, accounting for ∼5% of *EGFR*-mutant cases and exhibiting markedly diminished response rates to TKIs compared to classical mutations [5,9]. While previous studies have primarily focused on therapeutic efficacy [[44], [45], [46]], the TME characteristics driving this resistance remain elusive. Leveraging single-cell transcriptomic profiling of clinical FFPE specimens, we demonstrate that EGFR_double LUADs harbor a distinctive TME landscape. This niche is characterized by the enrichment of an EMT-like malignant (MP4) and metabolically reprogrammed alveolar macrophages, bridged by a COL4A3-CD44 interaction signaling. Our study provides the first single-cell resolution blueprint of the *EGFR* (G719X/S768I) TME, identifying a conserved metabolic-myeloid signaling niche that orchestrates the immunosuppressive milieu characteristic of this challenging clinical subtype.

Beyond its established role in driving metastasis and drug resistance [47], EMT appears inextricably linked to metabolic reprogramming. The EMT-like MP4 in both EGFR_double and Non_EGFR LUADs underscores a conserved malignant program characterized by profound lipid-EMT metabolic coupling. This synergy suggests a reciprocal reinforcement loop: EMT-induced lipid shifts likely stabilize the mesenchymal phenotype, thereby fostering a highly invasive and chemoresistant state [33]. Crucially, the preferential activation of O-glycan biosynthesis in EGFR_double suggests a specialized regulatory layer. Given that O-glycan modifications are pivotal for the glycosylation and proteostatic stability of *CD44* [34,35], this metabolic-glycosylation niche likely serves as a functional switch that enhances *CD44*-mediated signaling at the malignant-myeloid niche. This mutation-specific vulnerability not only defines the aggressive feature of EGFR_double, but also identifies a potential metabolic target to bypass therapeutic resistance.

The metabolically stressed, immunosuppressive phenotype of alveolar macrophages in EGFR_double aligns with the emerging paradigm of myeloid reprogramming as a driver of LUAD progression. While previous research has linked *TP53* mutations to the assembly of pro-metastatic *SPP1*^+^ macrophage-fibroblast niche [14], our findings suggest that macrophage plasticity represents a conserved adaptive mechanism across diverse genomic landscapes. Despite distinct subtype compositions between *EGFR* and *TP53*-mutant contexts, this functional convergence underscores myeloid subversion as a fundamental hallmark of TME. Targeting these mutation-specific myeloid states may therefore offer a potent strategy to overcome the immunosuppressive barriers inherent to different LUAD mutation types. Furthermore, Izumi et al. stablished cell-intrinsic mechanisms that *BCL2L1*-driven anti-apoptotic signaling in *EGFR*-mutant lung cancer [12], where our findings highlight the critical contribution of extrinsic microenvironmental factors to therapeutic recalcitrance. We propose that the *COL4A3-CD44* interaction serves as a specialized communication between EMT-like malignant cells and macrophages, fostering a robust immunosuppressive TME. This extrinsic signaling pathway likely operates in concert with cell-autonomous resistance programs, collectively underpinning the diminished TKI efficacy and aggressive clinical course observed in EGFR_double. The *COL4A3-CD44* interaction identified here represents a specialized molecular TME for malignant-myeloid crosstalk. CD44 is classically a receptor for hyaluronic acid and can activate TGF-β1 and EGF signaling pathways that drive EMT [48], which interact with COL4A3 work as a basement membrane component often hijacked during tumor progression [49,50], suggests a bidirectional signaling loop that reinforces both the mesenchymal state of MP4 and the immunosuppressive polarization of alveolar macrophages. This crosstalk likely contributes a pro-tumorigenic niche that complements cell-intrinsic TKI resistance. Given the rarity of *EGFR* G719X/S768I double-mutant LUAD, the small mIHC validation cohort (n = 11) may constrain the assessment of inter-individual heterogeneity. To address this, we validated the *COL4A3-CD44* association in three independent LUAD cohorts, where consistent positive correlations supported the robustness of the axis, although larger spatially resolved cohorts will be needed for further validation. Our drug sensitive analysis further identified that *EGFR* double-mutant LUADs were predicted to be sensitive to p38 MAPK [51], BET proteins [52], PARP [53], IGF-1R/IR [54], JAK/STAT signaling [55], and the TGF-β pathway [56]. Agents such as Amivantamab [57,58] and emerging antibody-drug conjugates (ADCs) targeting tumor-associated antigens, such as TROP2, may partially rely on effective tumor-immune engagement [59]. However, the immunosuppressive macrophage states and extracellular matrix-receptor signaling identified here, such as the *COL4A3-CD44* axis, may impair immune effector function and antigen accessibility, which potentially limiting the efficacy of these therapeutic modalities.

Recent studies have also emphasized immune evasion in shaping therapeutic response in non-small cell lung cancer. Notably, anti-phagocytic pathway such as CD24 have been implicated in suppressing macrophage-mediated tumor clearance [60,61]. These findings reinforce the notion that effective treatment of different EGFR-mutant LUAD may require co-targeting oncogenic signaling and TME-mediated immune escape.

## Conclusion

In summary, this study provides the first comprehensive single-cell transcriptomic characterization of the TME in *EGFR* G719X/S768I double-mutant LUAD, a clinically recalcitrant subtype characterized by diminished TKI responsiveness. By integrating FFPE and fresh samples of three *EGFR* mutation types, we identified a specialized EMT-like malignant subpopulation (MP4) that orchestrates of the tumor microenvironment niches. We demonstrate that a unique lipid-glycosylation metabolic niche stabilizes *CD44* expression, which in turn facilitates pathological crosstalk with metabolically reprogrammed alveolar macrophages via the *COL4A3*-*CD44* interaction signaling and in situ validation by mIHC. This metabolic-myeloid niche likely complements intrinsic resistance mechanisms to diminish TKI responsiveness. Furthermore, our work highlights the pivotal utility of FFPE tissues in resolving the TME of rare clinical cohorts. Several limitations of this study need consideration. First, limited sample size (n = 11) reflects the clinical rarity of the *EGFR* G719X/S768I double mutation. Second, FFPE-based snRNA-seq effectively captures epithelial and myeloid diversity, but with reduced T/NK cell detection [16], which limited our studies to investigate T and B cells. Third, although mIHC provided preliminary in situ validation of the *COL4A3-CD44* interaction, further functional validation is required. Moreover, our drug sensitivity analysis was based on computational predictions and should therefore be considered hypothesis-generating rather than direct experimental evidence. The current lack of established *EGFR* G719X/S768I double-mutant models limits functional validation, and patient-derived organoids (PDOs) or genetically engineered knock-in models will be important for validating the predicted vulnerabilities in future work [[62], [63], [64]]. FFPE tissues provide an important resource for characterizing tumor cell heterogeneity and uncovering mechanisms underlying treatment resistance [15,16,65].

Collectively, these findings identify the *COL4A3-CD44* associated metabolic-myeloid niche as a potential driver of TME-mediated therapeutic resistance and situate EGFR_double LUAD within the evolving landscape of antibody-based, ADC, and microenvironment-targeted therapeutic strategies.

## Ethics statement

This study was performed in accordance to the tenets of the Declaration of Helsinki, and the samples were collected with the Ethics Committee of Qujing First People’s Hospital (No. 2024– 076– 02). All the people and patients who participated in our study provided written consent before they started.

## Funding

This study was supported in part by Yunnan Provincial Academician Expert Workstation (202305AF150091), National Key Clinical Specialty Construction Project for Oncology, Yunnan Provincial Major Science and Technology Special Project (202302AA310039) to Chao Zhang; Yunnan Fundamental Research Projects (202401AY070001– 140) to Jialing Lv; the First-Class Discipline Team of Kunming Medical University (2024XKTDTS05), National Natural Science Foundation of China (82,573,122, 82,460,519) to Runxiang Yang; and Yunnan Basic Research Program (202401AY070001– 330) to Fang Yang.

## CRediT authorship contribution statement

**Chao Zhang:** Writing – review & editing, Methodology, Data curation. **Lin Liu:** Writing – review & editing, Writing – original draft, Methodology, Formal analysis. **Ruoyu Deng:** Writing – review & editing, Investigation, Data curation. **Jialing Lv:** Writing – review & editing, Data curation. **Chenghao Mei:** Writing – review & editing, Validation. **Wen Zhang:** Writing – review & editing, Data curation. **Liuqing Yang:** Writing – review & editing, Writing – original draft, Investigation. **Fang Yang:** Writing – review & editing, Data curation. **Runxiang Yang:** Writing – review & editing, Funding acquisition. **Yupeng Cun:** Writing – review & editing, Writing – original draft, Methodology, Investigation, Conceptualization.

## Declaration of competing interest

The authors declare that they have no known competing financial interests or personal relationships that could have appeared to influence the work reported in this paper.

## Data Availability

All the raw data involved in this study have been deposited at the National Genomics Data Center of China with BioProject accession: PRJCA050093. The data under accession PRJCA050093 will be available after published.

## Appendix Supplementary materials

Image, application 1

Image, application 2

Image, application 3

## References

[1] Sung H., Ferlay J., Siegel R.L, Laversanne M., Soerjomataram I., Jemal A., Bray F. (2021). Global Cancer statistics 2020: GLOBOCAN estimates of incidence and mortality worldwide for 36 cancers in 185 countries. CACancer J. Clin..

[2] Wakelee H.A, Chang E.T, Gomez S.L, Keegan T.H, Feskanich D., Clarke C.A, Holmberg L., Yong L.C, Kolonel L.N, Gould M.K, West D.W (2007). Lung cancer incidence in never smokers. J. Clin. Oncol..

[3] Devarakonda S., Li Y., Martins Rodrigues F., Sankararaman S., Kadara H., Goparaju C., Lanc I., Pepin K., Waqar S.N, Morgensztern D. (2021). Genomic profiling of lung adenocarcinoma in never-smokers. J. Clin. Oncol..

[4] Choong W.K, Sung T.Y (2021). Somatic mutation subtypes of lung adenocarcinoma in East Asian reveal divergent biological characteristics and therapeutic vulnerabilities. iScience.

[5] Harrison P.T, Vyse S., Huang P.H (2020). Rare epidermal growth factor receptor (EGFR) mutations in non-small cell lung cancer. Semin. Cancer Biol..

[6] Robichaux J.P, Le X., Vijayan R.SK., Hicks J.K, Heeke S., Elamin Y.Y, Lin H.Y, Udagawa H., Skoulidis F., Tran H. (2021). Structure-based classification predicts drug response in EGFR-mutant NSCLC. Nature.

[7] Beau-Faller M., Prim N., Ruppert A.M, Nanni-Metellus I., Lacave R., Lacroix L., Escande F., Lizard S., Pretet J.L, Rouquette I. (2014). Rare EGFR exon 18 and exon 20 mutations in non-small-cell lung cancer on 10 117 patients: a multicentre observational study by the French ERMETIC-IFCT network. Ann. Oncol..

[8] Morimoto T., Yamasaki K., Shingu T., Higashi Y., Maeda Y., Uryu T., Kubo N., Kawaguchi T., Nishida C., Yatera K. (2023). A rare case of double primary lung adenocarcinomas with uncommon complex EGFR G719X and S768I mutations and pleomorphic carcinoma. Thorac. Cancer.

[9] Chiu C.H, Yang C.T, Shih J.Y, Huang M.S, Su W.C, Lai R.S, Wang C.C, Hsiao S.H, Lin Y.C, Ho C.L (2015). Epidermal growth factor receptor tyrosine kinase inhibitor treatment response in advanced lung adenocarcinomas with G719X/L861Q/S768I mutations. J. Thorac. Oncol..

[10] Altorki N.K, Markowitz G.J, Gao D., Port J.L, Saxena A., Stiles B., McGraw T., Mittal V. (2019). The lung microenvironment: an important regulator of tumour growth and metastasis. Nat. Rev. Cancer.

[11] He D., Wang D., Lu P., Yang N., Xue Z., Zhu X., Zhang P., Fan G. (2021). Single-cell RNA sequencing reveals heterogeneous tumor and immune cell populations in early-stage lung adenocarcinomas harboring EGFR mutations. Oncogene.

[12] Izumi M., Fujii M., Kobayashi I.S, Ho V., Kashima Y., Udagawa H., Costa D.B, Kobayashi S.S (2024). Integrative single-cell RNA-seq and spatial transcriptomics analyses reveal diverse apoptosis-related gene expression profiles in EGFR-mutated lung cancer. Cell Death. Dis..

[13] Wu F., Fan J., He Y., Xiong A., Yu J., Li Y., Zhang Y., Zhao W., Zhou F., Li W. (2021). Single-cell profiling of tumor heterogeneity and the microenvironment in advanced non-small cell lung cancer. Nat. Commun..

[14] Zhao W., Nguyen T.T, Bhagwat A., Kumar A., Giotti B., Kepecs B., Weirather J.L, Mahadevan N.R, Segerstolpe A., Dolasia K. (2025). A cellular and spatial atlas of TP53-associated tissue remodeling defines a multicellular tumor ecosystem in lung adenocarcinoma. Nat. Cancer.

[15] Janesick A., Shelansky R., Gottscho A.D, Wagner F., Williams S.R, Rouault M., Beliakoff G., Morrison C.A, Oliveira M.F, Sicherman J.T (2023). High resolution mapping of the tumor microenvironment using integrated single-cell, spatial and in situ analysis. Nat. Commun..

[16] Trinks A., Milek M., Beule D., Kluge J., Florian S., Sers C., Horst D., Morkel M., Bischoff P. (2024). Robust detection of clinically relevant features in single-cell RNA profiles of patient-matched fresh and formalin-fixed paraffin-embedded (FFPE) lung cancer tissue. Cell Oncol..

[17] (2014). Cancer Genome Atlas Research N: comprehensive molecular profiling of lung adenocarcinoma. Nature.

[18] Chen J., Yang H., Teo A.SM., Amer L.B, Sherbaf F.G, Tan C.Q, Alvarez J.JS., Lu B., Lim J.Q, Takano A. (2020). Genomic landscape of lung adenocarcinoma in East Asians. Nat. Genet..

[19] Zhang H., Liu C., Li L., Feng X., Wang Q., Li J., Xu S., Wang S., Yang Q., Shen Z. (2021). Genomic evidence of lung carcinogenesis associated with coal smoke in Xuanwei area, China. Natl. Sci. Rev..

[20] Hao Y., Hao S., Andersen-Nissen E., Mauck W.M, Zheng S., Butler A., Lee M.J, Wilk A.J, Darby C., Zager M. (2021). Integrated analysis of multimodal single-cell data. Cell.

[21] Tirosh I., Izar B., Prakadan S.M, Wadsworth M.H, Treacy D., Trombetta J.J, Rotem A., Rodman C., Lian C., Murphy G. (2016). Dissecting the multicellular ecosystem of metastatic melanoma by single-cell RNA-seq. Science.

[22] L. Yerly, M. Andreatta, J. Garnica, C. Nardin, J.D Domizio, F. Aubin, M. Gilliet, S.J Carmona, F. Kuonen: Wounding triggers invasive progression in human basal cell carcinoma. 2025:2024.2005.2031.596823.

[23] Wu Y., Yang S., Ma J., Chen Z., Song G., Rao D., Cheng Y., Huang S., Liu Y., Jiang S. (2022). Spatiotemporal immune landscape of colorectal cancer liver metastasis at single-cell level. Cancer Discov..

[24] Aibar S., Gonzalez-Blas C.B, Moerman T., Huynh-Thu V.A, Imrichova H., Hulselmans G., Rambow F., Marine J.C, Geurts P., Aerts J. (2017). SCENIC: single-cell regulatory network inference and clustering. Nat. Methods.

[25] Bergen V., Lange M., Peidli S., Wolf F.A, Theis F.J (2020). Generalizing RNA velocity to transient cell states through dynamical modeling. Nat. Biotechnol..

[26] Hanzelmann S., Castelo R., Guinney J. (2013). GSVA: gene set variation analysis for microarray and RNA-seq data. BMC Bioinform..

[27] Ritchie M.E, Phipson B., Wu D., Hu Y., Law C.W, Shi W., Smyth G.K (2015). Limma powers differential expression analyses for RNA-sequencing and microarray studies. Nucleic. Acids. Res..

[28] Schubert M., Klinger B., Klunemann M., Sieber A., Uhlitz F., Sauer S., Garnett M.J, Bluthgen N., Saez-Rodriguez J. (2018). Perturbation-response genes reveal signaling footprints in cancer gene expression. Nat. Commun..

[29] Jin S., Guerrero-Juarez C.F, Zhang L., Chang I., Ramos R., Kuan C.H, Myung P., Plikus M.V (2021). Nie Q: inference and analysis of cell-cell communication using CellChat. Nat. Commun..

[30] Browaeys R., Saelens W., Saeys Y. (2020). NicheNet: modeling intercellular communication by linking ligands to target genes. Nat. Methods.

[31] Maeser D., Gruener R.F (2021). Huang RS: oncoPredict: an R package for predicting in vivo or cancer patient drug response and biomarkers from cell line screening data. Br. Bioinform..

[32] Liu J., Huang X., Chen C., Wang Z., Huang Z., Qin M., He F., Tang B., Long C., Hu H. (2023). Identification of colorectal cancer progression-associated intestinal microbiome and predictive signature construction. J. Transl. Med..

[33] Din Z.U, Cui B., Wang C., Zhang X., Mehmood A., Peng F., Liu Q. (2025). Crosstalk between lipid metabolism and EMT: emerging mechanisms and cancer therapy. Mol. Cell Biochem..

[34] Leon F., Seshacharyulu P., Nimmakayala R.K, Chugh S., Karmakar S., Nallasamy P., Vengoji R., Rachagani S., Cox J.L, Mallya K. (2022). Reduction in O-glycome induces differentially glycosylated CD44 to promote stemness and metastasis in pancreatic cancer. Oncogene.

[35] Du T., Jia X., Dong X., Ru X., Li L., Wang Y., Liu J., Feng G., Wen T. (2020). Cosmc disruption-mediated aberrant O-glycosylation suppresses breast cancer cell growth via impairment of CD44. Cancer Manag. Res..

[36] Shenoy U.S, Adiga D., Alhedyan F., Kabekkodu S.P, Radhakrishnan R. (2024). HOXA9 transcription factor is a double-edged sword: from development to cancer progression. Cancer Metastasis Rev..

[37] Acharya A., Baek S.T, Huang G., Eskiocak B., Goetsch S., Sung C.Y, Banfi S., Sauer M.F, Olsen G.S, Duffield J.S (2012). The bHLH transcription factor Tcf21 is required for lineage-specific EMT of cardiac fibroblast progenitors. Development.

[38] Aegerter H., Lambrecht B.N, Jakubzick C.V (2022). Biology of lung macrophages in health and disease. Immunity.

[39] Lavin Y., Kobayashi S., Leader A., Amir E.D, Elefant N., Bigenwald C., Remark R., Sweeney R., Becker C.D, Levine J.H (2017). Innate immune landscape in early lung adenocarcinoma by paired single-cell analyses. Cell.

[40] Li W., Zou Y., Zheng W., Zhang J., Cen C., Li R., Cheng H., Hou S., Wan W., Liang L. (2025). Transcription factor EGR2 drives cataract formation through IGFBP3-mediated oxidative injury in lens epithelial cells. Free Radical Biol. Med..

[41] Zhang D., Lin J., Chao Y., Zhang L., Jin L., Li N., He R., Ma B., Zhao W., Han C. (2018). Regulation of the adaptation to ER stress by KLF4 facilitates melanoma cell metastasis via upregulating NUCB2 expression. J. Exp. Clin. Cancer Res..

[42] Gao H., Guo R.F, Speyer C.L, Reuben J., Neff T.A, Hoesel L.M, Riedemann N.C, McClintock S.D, Sarma J.V, Van Rooijen N. (2004). Stat3 activation in acute lung injury. J. Immunol..

[43] Quail D.F, Joyce J.A (2013). Microenvironmental regulation of tumor progression and metastasis. Nat. Med..

[44] Pan X., Shi M. (2024). Successful therapy of a critically ill non-small cell lung cancer patient with compound mutations in EGFR G719X and S768I genes using furmonertinib: a case report. Heliyon.

[45] Kutsuzawa N., Takahashi F., Tomomatsu K., Obayashi S., Takeuchi T., Takihara T., Hayama N., Oguma T., Aoki T., Asano K. (2020). Successful treatment of a patient with lung adenocarcinoma harboring compound EGFR gene mutations, G719X and S768I, with Afatinib. Tokai. J. Exp. Clin. Med..

[46] Wang J.L, Fu Y.D, Gao Y.H, Li X.P, Xiong Q., Li R., Hou B., Huang R.S, Wang J.F, Zhang J.K (2022). Unique characteristics of G719X and S768I compound double mutations of epidermal growth factor receptor (EGFR) gene in lung cancer of coal-producing areas of East Yunnan in Southwestern China. Genes Env..

[47] Dong Y., Poon G.FT., Arif A.A, Lee-Sayer S.SM., Dosanjh M., Johnson P. (2018). The survival of fetal and bone marrow monocyte-derived alveolar macrophages is promoted by CD44 and its interaction with hyaluronan. Mucosal. Immunol..

[48] Li L., Qi L., Liang Z., Song W., Liu Y., Wang Y., Sun B., Zhang B., Cao W. (2015). Transforming growth factor-beta1 induces EMT by the transactivation of epidermal growth factor signaling through HA/CD44 in lung and breast cancer cells. Int. J. Mol. Med..

[49] Sun F., Chen H., Dai X., Hou Y., Li J., Zhang Y., Huang L., Guo B., Yang D. (2024). Liposome-lentivirus for miRNA therapy with molecular mechanism study. J. Nanobiotechnology.

[50] Jiang C.P, Wu B.H, Chen S.P, Fu M.Y, Yang M., Liu F., Wang B.Q (2013). High COL4A3 expression correlates with poor prognosis after cisplatin plus gemcitabine chemotherapy in non-small cell lung cancer. Tumour. Biol..

[51] Zhu N., Zhang X.J, Zou H., Zhang Y.Y, Xia J.W, Zhang P., Zhang Y.Z, Li J., Dong L., Wumaier G., Li S.Q (2021). PTPL1 suppresses lung cancer cell migration via inhibiting TGF-beta1-induced activation of p38 MAPK and Smad 2/3 pathways and EMT. Acta Pharmacol. Sin..

[52] Zhang X., Yang Y., Zou H., Yang Y., Zheng X., Corey E., Zoubeidi A., Mitsiades N., Yu A.M, Li Y., Chen H.W (2025). Effective therapeutic targeting of tumor lineage plasticity in neuroendocrine prostate cancer by BRD4 inhibitors. Acta Pharm. Sin.B.

[53] Kumar M., Jaiswal R.K, Prasad R., Yadav S.S, Kumar A., Yadava P.K, Singh R.P (2021). PARP-1 induces EMT in non-small cell lung carcinoma cells via modulating the transcription factors Smad4, p65 and ZEB1. Life Sci..

[54] Zhou Y., Zhang Z., Wang N., Chen J., Zhang X., Guo M., John Zhong L., Wang Q. (2018). Suppressor of cytokine signalling-2 limits IGF1R-mediated regulation of epithelial-mesenchymal transition in lung adenocarcinoma. Cell Death. Dis..

[55] Dakal T.C, Bhushan R., Xu C., Gadi B.R, Cameotra S.S, Yadav V., Maciaczyk J., Schmidt-Wolf I.GH., Kumar A., Sharma A. (2024). Intricate relationship between cancer stemness, metastasis, and drug resistance. MedComm.

[56] Wang X., Eichhorn P.JA., Thiery J.P (2023). TGF-beta, EMT, and resistance to anti-cancer treatment. Semin. Cancer Biol..

[57] Zhou F., Guo H., Xia Y., Le X., Tan D.SW., Ramalingam S.S, Zhou C. (2025). The changing treatment landscape of EGFR-mutant non-small-cell lung cancer. Nat. Rev. Clin. Oncol..

[58] Yoshichika R., Mukohara F., Yamada K., Nagasaki J., Watanabe H., Ueda Y., Suzawa K., Shien K., Toyooka S., Ishino T., Togashi Y. (2026). Immunological effects of amivantamab in EGFR or MET-expressing non-small cell lung cancer. Cancer Immunol. Immunther..

[59] Su P.L, Furuya N., Asrar A., Rolfo C., Li Z., Carbone D.P, He K. (2025). Recent advances in therapeutic strategies for non-small cell lung cancer. J. Hematol. Oncol..

[60] Liang J., Bi G., Huang X., Xu Z., Huang Y., Bian Y., Shan G., Guo W., Yan Y., Sui Q. (2025). CD24 is a promising immunotherapeutic target for enhancing efficacy of third-generation EGFR-TKIs on EGFR-mutated lung cancer. Cancer Commun. L.

[61] Wang G., Yuan S., Xu F., Mao D., Hu T., Xu Q., Li L., Wei Z., Yuan S. (2026). Radiotherapy synergizes with CD24 blockade to initiate macrophage-driven systemic antitumor immunity in non-small cell lung cancer. Exp. Hematol. Oncol..

[62] Wensink G.E, Elias S.G, Mullenders J., Koopman M., Boj S.F, Kranenburg O.W, Roodhart J.ML. (2021). Patient-derived organoids as a predictive biomarker for treatment response in cancer patients. NPJ. Precis. Oncol..

[63] Wang D., Villenave R., Stokar-Regenscheit N., Clevers H. (2026). Human organoids as 3D in vitro platforms for drug discovery: opportunities and challenges. Nat. Rev. Drug Discovery.

[64] Kim S.Y, Kim S.M, Lim S., Lee J.Y, Choi S.J, Yang S.D, Yun M.R, Kim C.G, Gu S.R, Park C. (2021). Modeling clinical responses to targeted therapies by patient-derived organoids of advanced lung adenocarcinoma. Clin. Cancer Res..

[65] Song J., Song X., Xie Y., Guo H., Cun Y. (2026). MIF-expressing tumor cells mediate immunotherapeutic resistance in esophageal squamous cell carcinoma. Theranostics.

