# Supplementary material for: A single-nucleus transcriptomic characterization of EGFR G719X/S768I double-mutant lung adenocarcinoma identifies an immunosuppressive niche defined by COL4A3-CD44 interaction
Source: Transl Oncol. 2026 Jul 31;72:102951. doi: 10.1016/j.tranon.2026.102951 (PMC13452252; doi:10.1016/j.tranon.2026.102951)
Supplement: Supplementary file 1 [file mmc1.pdf]

Ethics Committee of Qujing Central Hospital of Yunnan Province

Ethical Review Approval Letter

Approval Letter No.: Ethical Review 2024-076-02

**Project Name**	EGFR G719X+S768I Co-Mutations in NSCLC		
**Project Category**	Others		
**Research Institution**	Ethics Committee of Qujing Central Hospital of Yunnan Province		
**Research Department**	Oncology	**Principal Investigator**	Ruoyu Deng
**Review Type**	☒ Initial Review ☐ Re-review		
**Review Method**	☐ Meeting Review ☐ Emergency Meeting Review ☒ Expedited Review		
**Review Date**	2024.11.25	**Review Venue**	Ethics Committee
**Review Committee Members**	Qi Xie		
**Reviewed Documents (Including but Not Limited To)**<br>1. Application Form for Animal Welfare and Ethical Review<br>2. Researcher's Resume			
**Review Decision**	☒ Approved		
**Validity Period of Approval Letter**	Consistent with the research period of this project		
**Declaration**	The composition and working procedures of this Ethics Committee comply with the GCP principles and the requirements of relevant national regulations.		
Signature of Chairman (or Vice Chairman):			
Ethics Committee of Qujing Central Hospital of Yunnan Province (Seal)			
Date: 2024.11.25			
