# Supplementary material for: A single-nucleus transcriptomic characterization of EGFR G719X/S768I double-mutant lung adenocarcinoma identifies an immunosuppressive niche defined by COL4A3-CD44 interaction
Source: Transl Oncol. 2026 Jul 31;72:102951. doi: 10.1016/j.tranon.2026.102951 (PMC13452252; doi:10.1016/j.tranon.2026.102951)
Supplement: Supplementary file 2 [file mmc3.docx]

Supplementary Information for

**A single-nucleus transcriptomic characterization of *EGFR* G719X/S768I double-mutant lung adenocarcinoma identifies an immunosuppressive niche defined by *COL4A3*-*CD44* interaction**

Chao Zhang^1,3,^**^†^**, Lin Liu^2,^**^†^**, Ruoyu Deng^3^, Jialing Lv^3^, Chenghao Mei^2^, Wen Zhang^3^, Liuqing Yang^2^, Fang Yang^1^, Runxiang Yang^1,*^, Yupeng Cun^2,*^

**This file includes:**

Supplementary Methods

Supplementary Figures S1 to S9

**Other supplementary material for this manuscript includes the following:**

Supplementary Tables S1 to S13 (.xls)

## Supplementary Methods

**Single nucleus RNA sequencing for FFPE tissue**

Two to three FFPE tissue sections (~50 μm) were placed in 1.5 mL tubes, deparaffinized with xylene, and rehydrated through a graded ethanol series to water. Tissues were homogenized in lysis buffer A on ice, digested with lysis buffer B at 37°C, filtered through a 40 μm strainer, centrifuged, and debris removed. Nuclei were resuspended in wash buffer and assessed for integrity and concentration via fluorescence staining and trypan blue.

Single-nucleus suspensions, 10X barcode gel beads, and oil were respectively loaded into designated wells of the Chromium Next GEM Chip Q (1000418, 10X Genomics), and Gel Beads-in-Emulsion (GEMs) were generated using the Chromium X system (10X Genomics). Cell lysis and cDNA synthesis were performed using the Chromium Single Cell Fixed RNA Kit (1000474, 10X Genomics), followed by barcoded cDNA purification, PCR amplification, and SPRIselect-based clean-up. Following enzymatic fragmentation, tailing, and adapter ligation, indexing was completed using the Dual Index Kit TS Set A (1000251, 10X Genomics). Library concentrations were quantified by Qubit 4.0 (Thermo Fisher), pooled accordingly, and sequenced on the Illumina NovaSeq 6000 platform.

**Somatic mutation detection and transcriptomic data processing of the Xuanwei cohort**

For whole-exome sequencing (WES) data from the Xuanwei (XW) cohort, raw FASTQ files were first subjected to quality control and filtering to obtain high-quality clean reads. Clean reads were then aligned to the GRCh38 human reference genome using BWA-MEM (v0.7.10)[1]. The resulting SAM files were converted to BAM format using SAMtools (v0.1.19), followed by sorting and duplicate marking with Picard (v2.20.2)[2]. Base quality score recalibration (BQSR) and local realignment around insertion/deletion (InDel) sites were subsequently performed using GATK (v4.1.2)[3].

To minimize false-positive variant calls, somatic single-nucleotide variants (SNVs) and InDels were independently identified using MuTect, MuTect2[4], VarScan2[5], and Strelka2[6]. Variants detected by at least two of the three callers (MuTect2, VarScan2, and Strelka2) were retained as high-confidence somatic mutations for downstream analyses.

For transcriptomic profiling, raw mRNA sequencing FASTQ files from the XW cohort were aligned to the UCSC hg38 reference genome using STAR (v2.7.3a)[7] with default parameters. Gene annotation was performed based on GENCODE v38, and gene expression levels were quantified using RSEM[8] (v1.2.28). The resulting normalized gene expression matrix was used for subsequent analyses.

**Single nucleus RNA-seq data processing**

Fastq files from FFPE samples were processed with Cell Ranger multi (v7.0.0) for alignment and quantification against the GRCh38 human reference genome. The filtered outputs were used for downstream analyses. Quality control was performed in Seurat (v4.4.0) by evaluating total UMIs, detected genes, and mitochondrial content (Supplementary Fig. 1C), followed by DoubletFinder (v2.0.3) to remove doublets. Public datasets underwent identical processing. After QC, FFPE and public data were integrated, yielding 246, 086 cells for analysis.

For dataset integration, Seurat objects were split by sample, normalized, and 2,000 variable features were identified. Shared genes were used for scaling and PCA, followed by batch correction via reciprocal PCA (RPCA). Dimensionality reduction was tailored by cell type: 45 PCs (resolution 0.5) for the whole dataset; 30 PCs (0.5) for epithelial; 26 PCs (0.3) for myeloid; 20 PCs (0.3) for T, NK, B, and plasma cells; 48 PCs (0.1) for fibroblasts; and 45 PCs (0.3) for endothelial cells. Integrated data were visualized with UMAP, and cluster-specific DEGs were identified using FindAllMarkers. Cell types were annotated based on canonical markers and the top 10 DEGs per cluster.

Cells showing aberrant or mixed expression profiles were excluded. Specifically, we removed: epithelial cells expressing *CCL5*, *TRAC*, *CD2*, *C1QB*, or *APOC1*; myeloid cells with *SFTA2*, *SFTPC*, or *SCGB3A2*; T/NK cells with *SFTPB*, *PIGR*, or *VEGFA*; B/plasma cells with *IL32*, *CD2*, *APOC1*, *MSR1*, *FABP4*, *GZMB*, *LILRA4*, or *IL3RA*; fibroblasts with *AGER*, *RTKN2*, *SFTPB*, *NAPSA*, *ITLN1*, or *SLC4A4*; and endothelial cells with *CCL5*, *GZMA*, *C1QB*, *APOC1*, or *LYZ*.

**CNV-based malignant cell inference and clustering analysis**

InferCNV (v1.18.1) was used to infer malignant cells in single-cell(nucleus) transcriptomes by detecting large-scale chromosomal gains and losses. Fresh and FFPE datasets were analyzed separately, with B cells as the reference population. CNVs were computed with cutoff = 0.1 and analysis_mode = “samples”, enabling the HMM model for CNV state prediction and noise reduction.

To quantify CNV alterations, estimateCNV was applied to calculate each cell’s CNV score and its correlation with the reference. Thresholds based on the mean CNV score and correlation coefficient distinguished malignant from non-malignant cells (Fresh: score > 0.003, cor > 0.2; FFPE: score > 0.0015, cor > 0.12).

Malignant cells identified by InferCNV were clustered by CNV profiles. The CNV matrix was z-score normalized and hierarchically clustered using Euclidean distance and the ward.D2 method, yielding five clusters. Heatmaps were generated via ComplexHeatmap (v2.14.0), and relationships among CNV clusters, mutation subtypes, and MP modules were visualized with ggsankey.

**Pathway activity analysis**

Differential pathway analysis between the two groups was conducted using limma (v3.54.2) with significance thresholds: GO-BP, *p* < 0.01; KEGG, *p* < 0.05. Pathways were classified as upregulated, downregulated, or non-significant based on logFC, with non-significant ones excluded. Remaining pathways were ranked by t-statistic and visualized using ggplot2 (v3.5.1).

MSigDB database (https://www.gsea-msigdb.org/gsea/index.jsp) downloaded HALLMARK gene sets including TNFA_SIGNALING_VIA_NFKB, ALLOGRAFT_REJECTION, INFLAMMATORY_RESPONSE, and INTERFERON_GAMMA_RESPONSE to evaluate pathway activity in macrophage subtypes. Gene sets such as HYPOXIA, TGF_BETA_SIGNALING, ANGIOGENESIS, and EPITHELIAL_MESENCHYMAL_TRANSITION were used to assess activity in endothelial and fibroblast subsets. Pathway module scores were calculated using AddModuleScore function, which computes the difference between the average expression of target genes and that of 100 control genes for each cell. Violin plots were employed to visualize pathway activity across different cell subtypes, with stat_summary used to annotate group-wise medians.

PROGENy (v1.24.0) was used to infer and standardize pathway activity scores across various cell types. Standardized scores were averaged within each cell cluster to obtain mean pathway activity per subtype, visualized using the package.

**Survival analysis**

Using TCGA LUAD and EAS datasets, survival analysis was performed with survival (v3.7-0)[9] and survminer (v0.4.9)[10] employing Kaplan-Meier method. Samples were stratified into high and low expression groups based on enrichment scores. Log-rank test p-value was calculated using survdiff function, and survival curves were visualized via ggsurvplot.

## Supplementary Figures

**Fig. S1. Batch effect removal and epithelial subcluster annotation.**

**Fig. S2. Copy number variation, malignant cell modules, and pathway activity.**

**Fig. S3. Identification, annotation and functional analysis of myeloid subtypes.**

**Fig. S4. Functional analysis of malignant functional modules and macrophages in EGFR_double and Non_EGFR LUAD.**

**Fig. S5. Functional co-localization validation of CD68, CD44, COL4A3, and PanCK, as well as their merged signals, in EGFR_double lung adenocarcinoma tissues.**

**Fig. S6. Functional co-localization validation of CD68, CD44, COL4A3, and PanCK, as well as their merged signals, in Non_EGFR lung adenocarcinoma tissues.**

**Fig. S7. Correlation analysis between *COL4A3* and *CD44* expression across independent LUAD cohorts.**

**Fig. S8. Drug sensitivity analysis.**

**Fig. S9. Identification and characterization of fibroblast and endothelial cell subtypes for different *EGFR* mutation type in LUAD**.

**Fig.S1. Batch effect removal and epithelial subcluster annotation.**

A. UMAP of LUAD samples before (left) and after (right) batch effect correction. B. UMAP plots showing batch effect correction across Adjacent, EGFR_single, EGFR_double, and Non_EGFR (before on the left, after on the right). C. Visualization of violin plots by sample category nFeature_RNA, nCount_RNA, and percent.mt after quality control. D. UMAP of epithelial subtypes. E. Marker genes used for epithelial subtype annotation. Dot size represents the proportion of cells expressing the gene; color intensity reflects average expression level.

**Fig. S2. Copy number variation, malignant cell modules, and pathway activity.**

A. Heatmaps of copy number variation profiles in epithelial subtypes using B cells as reference, for both FFPE and fresh samples. Red indicates copy number gains, blue indicates losses. B. Functional modules of malignant cells characterized by HALLMARK pathway signatures. C. Functional modules of malignant cells characterized by GO-BP pathway signatures. D. Survival analysis based on malignant module infiltration scores using TCGA and EAS dataset. "High" and "Low" indicate high and low infiltration scores, respectively; p-value calculated via log-rank test. E. UMAP visualization of malignant cell modules colored by module identity (left), and bar plots showing the distribution of modules across EGFR_single, EGFR_double, and Non_EGFR (right). F. Average pathway activity scores across different malignant cell modules.

**Fig. S3. Identification, annotation and functional analysis of myeloid subtypes.**

A. UMAP of myeloid subtypes, color-coded by cell type. B. Marker genes used for annotation of myeloid subtypes. Dot size indicates the proportion of expressing cells, color intensity reflects average expression level. C. GSVA-based comparison of KEGG pathway activities in Alveolar_Macro between EGFR_double and Non_EGFR groups. Khaki indicates EGFR_double and blue indicates Non_EGFR. *p < 0.05, ***p < 0.001. D. Survival analysis based on Alveolar_Macro infiltration scores in EGFR_double and Non_EGFR using EAS data. "High" and "Low" denote high and low infiltration scores; p-values were calculated using the log-rank test. E. Survival analysis of AM infiltration scores in EGFR_double and Non_EGFR based on TCGA data. High and Low denote high and low infiltration, respectively. P-values were calculated using the log-rank test. F. Activity scores of Alveolar_Macro and CXCL10⁺ macrophages across Adjacent, EGFR_single, EGFR_double, and Non_EGFR, with dots representing median values. G. *CD44* expression in Myeloid subpopulations (top) and classification by EGFR_double and Non_EGFR expression patterns (bottom).

**Fig. S4. Functional analysis of malignant functional modules and macrophages in EGFR_double and Non_EGFR LUAD.**

A. Scatter plots showing the functional roles of cell subytpes within the cell-cell communication networks of EGFR_double and Non_EGFR groups. The x- and y-axes represent the outgoing and incoming signaling strengths, respectively. Each dot denotes a cell population, with size indicating its relative involvement in the communication network. B. Downregulated ligand-receptor pairs in EGFR_double; circle color indicates interaction probability between cell populations (redder means higher), and circle size represents statistical significance (p-value). C. Expression of *COL4A3* and *CD44* across myeloid subtypes and malignant functional modules, with dot size indicating median expression levels. D. Expression levels of *COL4A1* and *SDC4* in myeloid subtypes and malignant functional modules (top), and in EGFR_double versus Non_EGFR (bottom), with each dot representing median values. E. The x-axis indicates the NicheNet-predicted regulatory potential of COL4A3, while the y-axis represents the correlation between target gene expression and *CD44* expression. The top 10 target genes are highlighted and labeled. F. Survival analysis based on COL4A3-CD44-mediated gene signature scores in EGFR_double from the EAS and XW datasets. High and Low denote groups with high and low gene signature scores, respectively.

**Fig. S5. Functional co-localization validation of CD68, CD44, COL4A3, and PanCK, as well as their merged signals, in EGFR_double LUAD tissues.**

Expression and colocalization of CD68, CD44, COL4A3, and PanCK, as well as their merged signals, in EGFR_double LUAD tissues.

**Fig. S6. Functional co-localization validation of CD68, CD44, COL4A3, and PanCK, as well as their merged signals, in Non_EGFR LUAD tissues.**

Expression and co-localization of CD68, CD44, COL4A3, and PanCK, as well as their merged signals, in Non_EGFR LUAD tissues.

**Fig. S7. Correlation analysis between COL4A3 and CD44 expression across independent LUAD cohorts.**

A. Spearman correlation analysis of COL4A3 and CD44 expression in the TCGA-LUAD (n=513), EAS (n=172), and Xuanwei (XW; n=150) cohorts using normalized gene expression matrices. B. Correlation analysis restricted to patients harboring EGFR double mutations (EGFR_double) in the EAS (n=14), TCGA (n=11), and XW (n=22) cohorts. C. Correlation analysis specifically performed in patients with EGFR G719X/S768I co-mutations. The left panel includes available cases from EAS (n=1), TCGA (n=2), and XW (n=15) cohorts after batch correction using ComBat. The right panel includes EAS (n=1) and XW (n=15) cases after ComBat correction. All correlations were evaluated using Spearman’s rank correlation test.

**Fig. S8. Drug sensitivity analysis.**

A. Correlation between *CD44* expression and IC50 to the drugs Doramapimod_1042, PRIMA-1MET_1131, Niraparib_1177, Linsitinib_1510, OTX015_1626, PRT062607_1631, AZD5153_1706, JAK1_8709_1718, LY2109761_1852, NVP-ADW742_1932, and BMS-754807_2171, calculated using Spearman’s method. B. Correlation between *COL4A3* expression and IC50 to the drugs Doramapimod_1042, BMS-536924_1091, Niraparib_1177, Linsitinib_1510, AZD5153_1706, JAK1_8709_1718, LY2109761_1852, BMS-754807_2171, and JQ1_21727, calculated using Spearman’s method. C. Venn diagram showing seven drugs associated with sensitivity to both *CD44* and *COL4A3* expression.

**Fig. S9. Identification and characterization of fibroblast and endothelial cell subtypes for different *EGFR* mutation type in LUAD.**

A. UMAP of re-clustering endothelial subtypes, colored by cell subtype. B. Marker genes used for endothelial subtypes annotation. Dot size represents the proportion of expressing cells, color intensity reflects average expression level. C. UMAP of re-clustering fibroblast subtypes, color-ed by cell type. D. Marker genes used for fibroblast subtypes annotation. Dot size represents the proportion of expressing cells, color intensity reflects average expression level.

**Supplementary Tables**

**Table S1. Clinical information of *EGFR* double mutant and No *EGFR* mutation LUAD patients**

**Table S2: Clinical information of public LUAD patients**

**Table S3. Statistics of FFPE snRNA-seq**

**Table S4. Sample information for EGFR_double-mutant cases from the EAS and XW cohorts (Bulk RNA-seq)**

**Table S5. GO-BP analysis of malignant cell modules**

**Table S6. Hallmark analysis of malignant cell modules**

**Table S7. Genes of malignant cell modules**

**Table S8. Alveolar macrophage EGFR_double and Non_EGFR GSVA analysis** **(GO-BP)**

**Table S9. Alveolar macrophage EGFR_double and Non_EGFR GSVA analysis** **(KEGG)**

**Table S10. Number of CD68+ cells and total number of co-localized signals in multiplex immunohistochemistry per sample**

**Table S11. Percentage of CD68+ cells relative to the total number of co-localized signals in multiplex immunohistochemistry**

**Table S12. CD44 and predicted drug data in EGFR_double samples (G719X/S768I)**

**Table S13. COL4A3 and predicted drug data in EGFR_double samples (G719X/S768I)**

**Reference**

1.	Li H: **Aligning sequence reads, clone sequences and assembly contigs with BWA-MEM.** *arXiv preprint arXiv* 2013**:**1303.3997.

2.	Poplin R, Ruano-Rubio V, DePristo MA, Fennell TJ, Carneiro MO, Van der Auwera GA, Kling DE, Gauthier LD, Levy-Moonshine A, Roazen D, et al: **Scaling accurate genetic variant discovery to tens of thousands of samples.** 2018**:**201178.

3.	DePristo MA, Banks E, Poplin R, Garimella KV, Maguire JR, Hartl C, Philippakis AA, del Angel G, Rivas MA, Hanna M, et al: **A framework for variation discovery and genotyping using next-generation DNA sequencing data.** *Nat Genet* 2011, **43:**491-498.

4.	Benjamin D, Sato T, Cibulskis K, Getz G, Stewart C, Lichtenstein L: **Calling Somatic SNVs and Indels with Mutect2.** 2019**:**861054.

5.	Koboldt DC, Zhang Q, Larson DE, Shen D, McLellan MD, Lin L, Miller CA, Mardis ER, Ding L, Wilson RK: **VarScan 2: somatic mutation and copy number alteration discovery in cancer by exome sequencing.** *Genome Res* 2012, **22:**568-576.

6.	Cibulskis K, Lawrence MS, Carter SL, Sivachenko A, Jaffe D, Sougnez C, Gabriel S, Meyerson M, Lander ES, Getz G: **Sensitive detection of somatic point mutations in impure and heterogeneous cancer samples.** *Nat Biotechnol* 2013, **31:**213-219.

7.	Dobin A, Davis CA, Schlesinger F, Drenkow J, Zaleski C, Jha S, Batut P, Chaisson M, Gingeras TR: **STAR: ultrafast universal RNA-seq aligner.** *Bioinformatics* 2013, **29:**15-21.

8.	Li B, Dewey CN: **RSEM: accurate transcript quantification from RNA-Seq data with or without a reference genome.** *BMC Bioinformatics* 2011, **12:**323.

9.	Therneau TM: **survival: A Package for Survival Analysis in R.** CRAN; 2020.

10.	Kassambara A, Kosinski M, Biecek P: **survminer: Drawing Survival Curves using ggplot2.** GitHub; 2025.
